# Evaluation of bifurcation stenting techniques at Catharina Hospital, Eindhoven in 2013

**DOI:** 10.1007/s12471-016-0911-x

**Published:** 2016-10-26

**Authors:** S. J. L. Leus, E. van Hagen, F. M. Zimmermann, L. X. van Nunen, M. van ‘t Veer, J. Koolen, N. H. J. Pijls

**Affiliations:** Department of Cardiology, Catharina Hospital, Eindhoven, The Netherlands

**Keywords:** Percutaneous coronary intervention, Bifurcation, Stenting, Culotte, Crush, Provisional stenting, T-stenting

## Abstract

**Aims:**

Percutaneous coronary intervention (PCI) of bifurcation lesions can be performed using various techniques. The aim of this study was to analyse the outcome of various techniques of bifurcation stenting in all patients undergoing bifurcation stenting at one large intervention centre in 2013, taking into account that more complex lesions might more often warrant a two-stent technique.

**Methods and results:**

This retrospective study included 260 consecutive patients who underwent non-primary PCI of a bifurcation lesion at the Catharina Hospital, Eindhoven, in 2013. Patients were classified into two groups: one-stent technique (provisional stenting), and two-stent techniques (culotte, crush and T‑stenting). The primary endpoint was the rate of restenosis at 1 year. The secondary endpoints were procedural complications (side branch occlusion, periprocedural infarction, and death) and major adverse cardiac events (MACE) at 1 year. Periprocedural complications occurred in 15 patients (5.8 %) with no difference between the groups (*p* = 0.27). After 1 year, restenosis occurred in 3.2 % of the patients in the one-stent technique group and 7.3 % in the two-stent technique group (*p* = 0.20). MACE at 1 year did not differ between the groups at 11.9 % and 12.2 % respectively (*p* = 1.00).

**Conclusions:**

This study shows that there is no significant difference between restenosis rate, or any other outcome parameter, with the different techniques of bifurcation stenting. Since provisional stenting is the simplest, most straightforward and cheapest approach, if technically feasible this technique has our preference as the initial approach, and an upgrade can be considered if the result is insufficient.

## Introduction

Approximately 15 % of coronary stenoses are located at a bifurcation [1]. Bifurcation lesions of epicardial coronary arteries can be treated using several techniques [2]. Percutaneous coronary intervention (PCI) with involvement of a bifurcation lesion is associated with a worse long-term outcome when both the main branch (MB) and side branch (SB) are stented [3]. Recent studies show that provisional stenting (using one stent for the MB only and ballooning the SB) is still the recommended technique [4, 5]. Nevertheless, in one-third of cases in which patients are scheduled for provisional stenting in the MB, it is necessary to implant a second stent in the SB [4]. One of the causes is low flow or occlusion of the SB, which occurs in approximately 8 % of lesions [6]. The effect of final kissing ballooning is also the subject of debate, in some studies it seems to be ineffective in provisional stenting [7, 8], but in other studies it is found to be superior when compared with culotte, crush and T‑stenting [9]. The usefulness of bioresorbable stents in bifurcation lesions [10, 11], even as coronary stent of choice in patients with stable angina pectoris and patients with unstable angina pectoris, has been discussed previously [12].

The aim of the present study was to investigate outcome using the various techniques of bifurcation stenting and to evaluate 1‑year outcome in all consecutive patients in whom bifurcation stenting was performed at one high volume referral centre in 2013.

## Methods

### Patient population

This retrospective study was performed at the Catharina Hospital in Eindhoven, the Netherlands. All consecutive patients who underwent a non-primary PCI (non-PPCI) of a de novo coronary bifurcation stenosis during 2013 were analysed. In this study, a procedure was defined as bifurcation stenting if all of the following four conditions were fulfilled: 1) the lesion should be a de novo lesion, 2) if one branch is occluded, an attempt should be made to open it, 3) the diameter of the SB should be larger than or equal to 1.5 mm, and 4) the stent could not be placed in the MB without involving the SB, or vice versa.

**Fig. 1:**
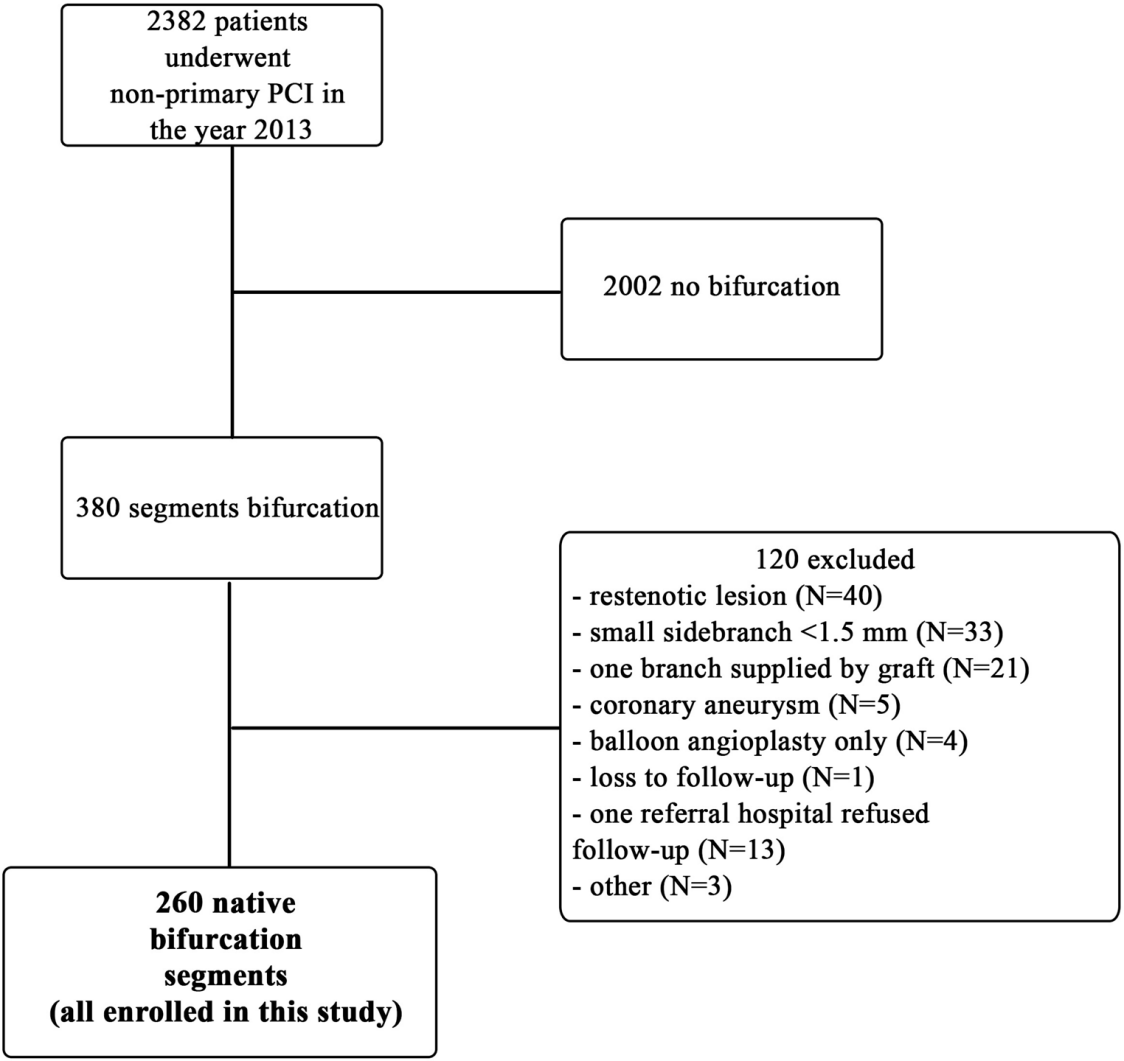
Flowchart of the study. *PCI* percutaneous coronary intervention

For this purpose, we reviewed all 2382 non-PPCI procedures performed in 2013. Of these patients, 2002 clearly did not have a bifurcation lesion. Of the 380 possible bifurcations, 260 were native bifurcation segments. Of these, 116 bifurcation segments were excluded because they were restenotic (*n* = 40), the SB was narrower than 1.5 mm (*n* = 33), one branch was supplied by a graft (*n* = 21), there was a combination with a coronary aneurysm (*n* = 5), only balloon angioplasty had been performed (*n* = 4), lost to follow-up (*n* = 1), refusal of one referring hospital to provide data (*n* = 13), or other (*n* = 3) (Fig. 1).

**Fig. 2:**
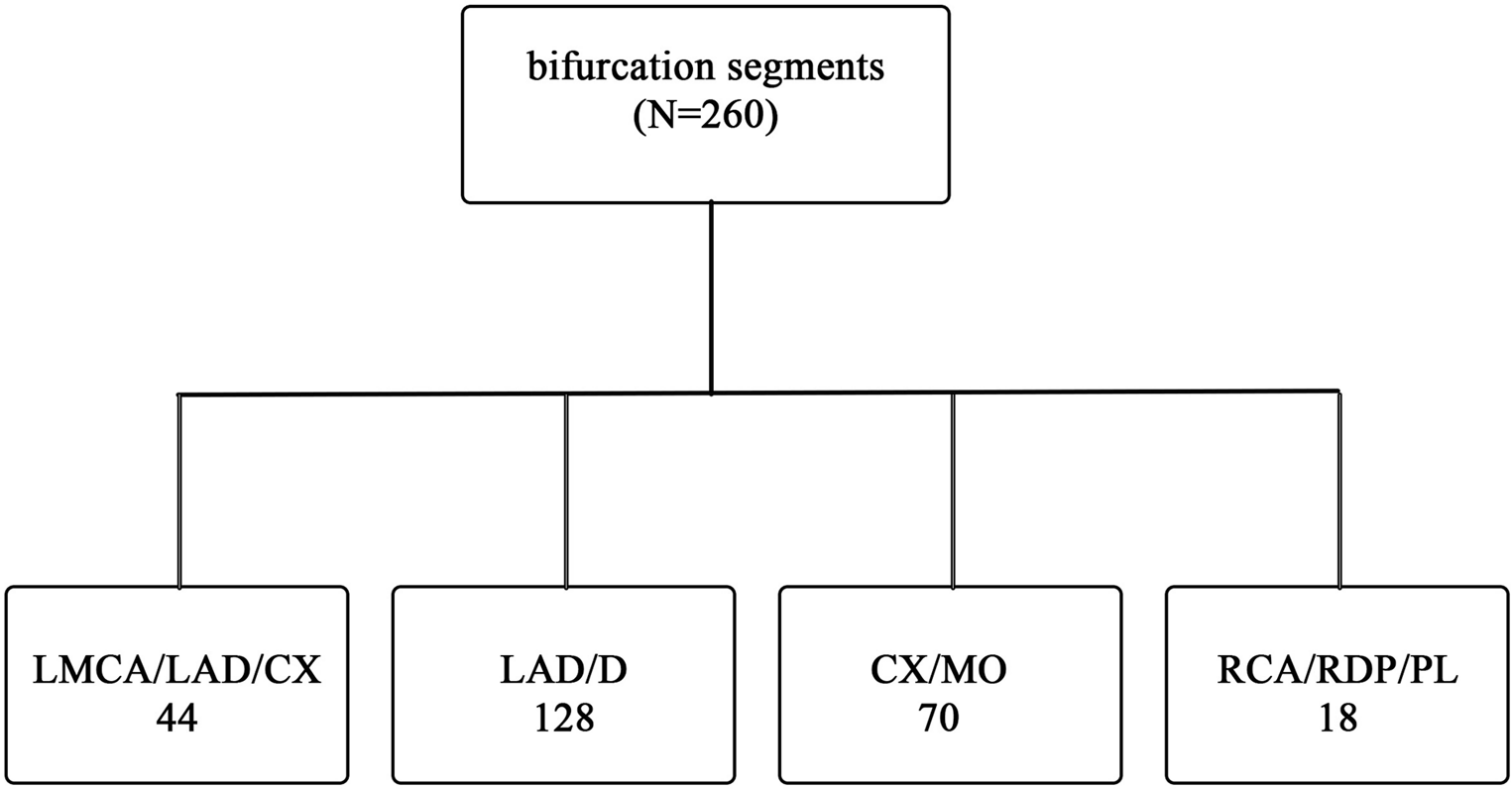
Bifurcation lesions according to location in the coronary tree. *CX* circumflex, *D* diagonal, *LAD* left anterior descending, *LMCA* left main coronary artery, *MO* margo obtusus, *PL* posterolateral, *RCA* right coronary artery, *RDP* ramus descending posterior

Patients were classified into different groups and subdivided with respect to location of the lesion, Medina classification and the technique of stenting ([13]; Tables 1 and 2, Fig. 2).

**Table 1:** Baseline characteristics

	One-stent technique (*n* = 219)	Two-stent techniques (*n* = 41)	*p*-value
Age (years)	67.4 ± 11.40	65.7 ± 12.3	0.23
Male	155 (70.8)	34 (82.9)	0.13
BMI (kg/m^2^)	27.6 ± 4.52	26.9 ± 5.3	0.45
Smoker	86 (39.3)	14 (34.1)	1.00
Diabetes	44 (20.1)	9 (22.0)	0.83
Family history of CAD	96 (43.8)	17 (41.5)	0.86
Dislipidaemia	119 (54.3)	21 (51.2)	0.74
Hypertension	132 (60.3)	22 (53.7)	0.49
Previous PCI	54 (24.7)	11 (26.8)	0.84
Previous CABG	16 (7.3)	2 (4.9)	0.75

Values are mean ±SD, *N* (%)

*BMI* body mass index, *CABG* coronary artery bypass grafting, *CAD* coronary artery disease, *PCI* percutaneous coronary intervention

**Table 2:** Angiographic characteristics

	One-stent technique (*n* = 219)	Two-stent techniques (*n* = 41)	*p*-value
Target vessel	–	–	0.50
LAD/D	106 (48.4)	22 (53.7)	–
CX/MO	57 (26.0)	13 (31.7)	–
LMCA/LAD/CX	40 (18.3)	4 (9.8)	–
RCA/RDP/PL	16 (7.3)	2 (4.9)	–
Vessel diameters	–	–	–
Proximal diameter MB (mm)	3.2 ± 0.62	3.2 ± 0.57	0.84
Distal diameter MB (mm)	3.1 ± 0.60	3.0 ± 0.48	0.68
SB diameter (mm)	2.2 ± 0.54	2.6 ± 0.55	<0.001
Medina classification	–	–	<0.001
True bifurcations	119 (54.3)	41 (100.0)	–
Non-true bifurcations	100 (45.7)	0 (0.0)	–

Values are mean ±SD, *N* (%)

*CX* circumflex, *D* diagonal, *LAD* left anterior descending, *LMCA* left main coronary artery, *MB* main branch, *MO* margo obtusus, *PL* posterolateral, *RCA* right coronary artery, *RDP* ramus descending posterior, *SB* side branch

True bifurcations refer to Medina classification 1‑1-1; 1‑0-1 and 0‑1-1, whereas non-true bifurcations refer to Medina classification 1‑1-0; 1‑0-0; 0‑1-0 and 0‑0-1

### Bifurcation stenting techniques

**Fig. 3:**
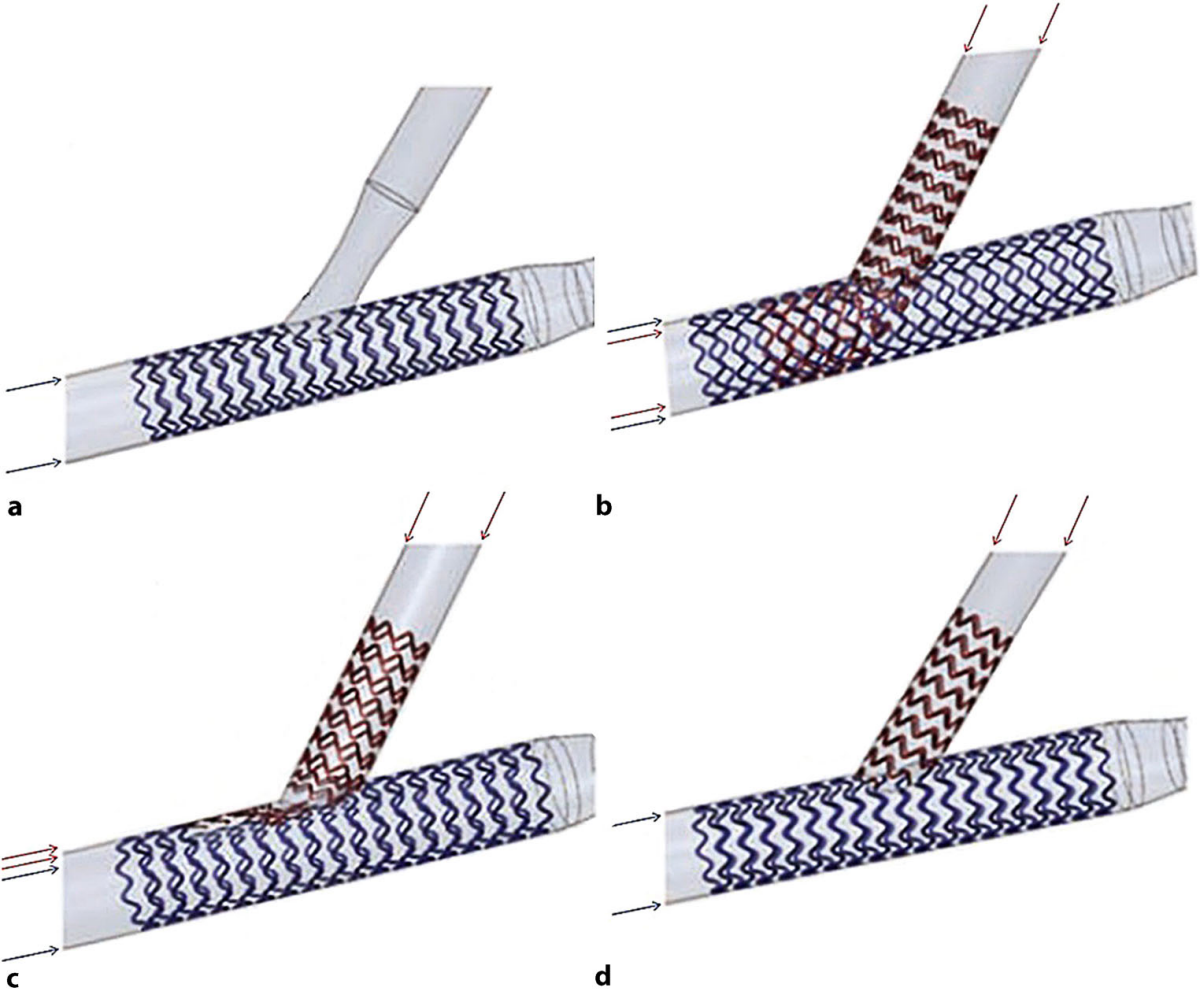
Techniques of stenting. Provisional (**a**), culotte (**b**), crush (**c**) and T‑stenting (**d**). The *blue arrows* represent the layer of struts of the main branch stent and the *red arrows* represents the layer of struts of the side branch stent

In provisional stenting it may be decided to stent the MB only without any intervention in the SB if there is a good thrombolysis in myocardial infarction (TIMI) flow (Fig. 3a). If TIMI flow is too low or there has been a significant plaque shift from the MB to the SB, a two-stent technique should be considered.

Culotte stenting consists of implanting a stent from the proximal MB to the distal MB. Then a second stent is placed from the proximal MB towards the SB through the struts of the first stent. Other operators prefer to stent the SB first and then the MB. This results in a double layer of struts in the proximal MB and presence of struts in the SB (Fig. 3b).

Crush stenting consists of advancing two stents simultaneously into both the MB and SB. The proximal segment of the SB stent is first deployed in the MB, and then it is crushed against the wall of the main vessel during deployment of the MB stent. Crush stenting results in a triple layer of struts in the proximal MB (Fig. 3c).

In T‑stenting, one stent is implanted in the MB and one stent at the SB. In this technique, there is no strut overlap at any site of the bifurcation. The struts at the orifice of the SB are intentionally removed. This technique is only possible if the SB branches off at an angle of >45º (Fig. 3d).

### Procedures

All procedures were performed according to the usual routine, either by the femoral or the radial approach. The choice of treatment technique was either discussed by the Heart Team prior to the procedure, or left to the discretion of the operator. Second generation drug eluting stents (DES) were used wherever possible and dual antiplatelet therapy was recommended for at least 1 year. Treatment and follow-up were in accordance with normal routine.

### Endpoints and follow-up

The primary endpoint was the rate of restenosis at 1 year, defined as >50 % stenosis on visual estimation in one of the branches at follow-up angiography, driven by recurrent angina pectoris or acute coronary syndrome. Secondary endpoints were MACE at 1 year (defined as the composite of death, myocardial infarction (MI) and target vessel revascularisation (TVR) by re-PCI or coronary artery bypass grafting (CABG)) at 1 year, the individual components of MACE, procedural complications such as SB occlusion, failure to dilate or to stent one of the branches, periprocedural infarction (>3 times the normal range of CK-MB or troponin-T) or death (Table 3). Angiography was only performed in the event of recurring symptoms.

**Table 3:** Periprocedural complication rate, 1‑year restenosis rate and MACE at 1 year comparing the one- versus two-stent techniques

	One-stent technique (*n* = 219)	Two-stent techniques (*n* = 41)	*p*-value
Periprocedural complication rate	–	–	0.27
Death	2 (0.9)	1 (2.4)	–
MI	8 (3.7)	3 (7.3)	–
TVR	1 (0.5)	0 (0.0)	–
1-year restenosis rate	7 (3.2)	3 (7.3)	0.20
MACE at 1 year	–	–	1.00
Death	12 (5.5)	3 (7.3)	–
MI	6 (2.7)	1 (2.4)	–
TVR	12 (5.5)	3 (7.3)	–

Values are *N* (%)

*MACE* major adverse cardiac events, *MI* myocardial infarction, *TVR* target vessel revascularisation

### Statistical analysis

Baseline characteristics were compared depending on normal distribution. Continuous variables were analysed using an unpaired T‑test and Mann-Whitney U test. Continuous variables are expressed as mean ± 1 SD and dichotomous variables are expressed as absolute numbers and percentages (%). Categorical variables were analysed with a chi-square test or Fisher’s exact test if appropriate. A *p*-value less than 0.05 was considered significant, and applicable tests were always two-sided. All analyses were conducted using SPSS 22.0 software (IBM corporation, Armonk, NY).

## Results

### Baseline characteristics and angiographic findings

A total of 260 patients who underwent a bifurcation stenosis PCI at the Catharina Hospital in 2013 and fulfilled the inclusion criteria were enrolled (Fig. 1).

Mean age was 67 ± 11 years; 189 men (73.0 %) and 71 women (27.0 %) were included. Of the 260 bifurcations there were 128 LAD/D, 70 CX/MO, 44 LMCA/LAD/CX and 18 RCA/RDP/PL segments involved (Fig. 2). Patients were divided into two groups according to the technique used (one-stent technique versus two-stent techniques). There were no significant differences in baseline characteristics (age, sex, risk factors and medical history), nor in the diameter of the proximal and distal MB (*p* = 0.84 and *p* = 0.68). The diameter of the SB was significantly larger in the two-stent technique group (*p* < 0.001). The two-stent technique group was more often classified as Medina 1‑1-1 when compared with other techniques (*p* < 0.001) (Tables 1 and 2).

### Procedural characteristics

A total of 219 patients were treated by the one-stent technique and 41 by a two-stent technique (20 by culotte, 13 by crush and 8 by T‑stenting). Eleven periprocedural events (5.0 %) occurred in the one-stent technique group, and four (9.8 %) in the two-stent technique group (*p* = 0.27).

### Restenosis rate at 1 year

Symptomatic restenosis occurred in 10 patients (3.8 %). Of these, 7 patients (3.2 %) were treated by the one-stent technique and 3 (7.3 %) by a two-stent technique (*p* = 0.20) (Table 3).

### MACE at 1 year

MACE occurred in 26 patients (11.9 %) in the one-stent technique versus 5 patients (12.2 %) in the group using a two-stent technique (*p* = 1.00) (Table 3). Fifteen patients (5.8 %) died within 1 year of follow-up: 12 (5.5 %) of them in the one-stent technique group and 3 (7.3 %) in the two-stent technique group (*p* = 0.71). Of these patients, 12 died of a cardiac cause: 9 in the one-stent technique group and 3 in the two-stent technique group (*p* = 0.41). Late MI occurred in 6 patients (2.7 %) in the one-stent technique group versus 1 in the two-stent technique group (2.4 %, *p* = 1.00). No significant difference was found in the rates of TVR comparing the one-stent versus the two-stent techniques (5.5 % vs 7.3 %, *p* = 0.71).

## Discussion and limitations

This study shows that in our population of 260 consecutive patients undergoing bifurcation stenting in a de novo lesion, there is no difference in restenosis rate at 1 year between provisional stenting (one-stent technique) and two-stent techniques. Neither did we find any differences in other adverse events such as procedural complications and total MACE at 1 year.

Our findings are not completely in accordance with other bifurcation studies. Over recent years several studies have compared bifurcation stenting techniques with mutually conflicting results. Comparing our results with those of other studies should be done with caution due to differences in baseline characteristics. Hildick-Smith et al. showed that two-stent techniques resulted in higher rates of MACE both in-hospital and at 9 months [5]. However, the inclusion and exclusion criteria in their study were different from those in our study. SB diameter had to be bigger than in our study and the left main coronary artery was excluded.

The DKCRUSH II trial did not show a difference in MACE between provisional stenting and crush stenting, but did show a significantly higher rate of TVR in the provisional group (*p* = 0.017) [18]. In our study, we did not find a significant difference in MACE between provisional (one-stent technique) and non-provisional stenting (two-stent techniques).

In our study, provisional stenting was mostly preferred and SB was not stented if not necessary. This is in accordance with recent consensus of the European Bifurcation Club. They recommend MB stenting with provisional SB treatment for the majority of bifurcation lesions, whereas only a large SB with significant ostial disease is likely to require a two-stent strategy [2]. Also the angle between MB and SB is sometimes used to determine which strategy is used [14].

The Nordic Bifurcation Study showed that two-stent techniques are associated with a longer procedure, requiring more fluoroscopy time and volume of contrast, and associated with a greater rate of procedure-related biomarker release [15].

Zamani et al. and Colombo et al. showed superiority of stenting an SB with DES. In their study, TVR, deaths and myocardial infarctions occurred less often in SB stenting with DES than with bare-metal stents [16, 17].

There are some specific strengths of this study that should be mentioned. In the first place, this study was performed within one single high volume centre at which 2382 non-PPCIs were performed in 1 year. Second, all consecutive patients were enrolled without a single exception and had a complete follow-up of 1 year. Third, almost all characteristics of the patients were complete. Fourth, there was no difference in baseline characteristics which ensures that our results are not influenced by selective inclusion of patients in either of the groups and the data at follow-up were almost completely available.

Our study has also some limitations. First, because of the retrospective nature of this study, it was not always possible to determine which of the patients treated by a two-stent technique were initially planned to be treated so and who underwent a two-stent technique after failure to achieve a satisfying result by provisional stenting [19]. Second, angiographic follow-up was driven by angina pectoris and acute coronary syndrome and the rates of restenosis and MACE were small. Third, in this population only a minority of patients were treated with a two-stent technique. Significant difference in procedural complications and restenosis at follow-up can therefore be missed due to underpower. Finally, where baseline characteristics are concerned, it can be suggested that the anatomy in the two-stent group was on average more complex than in the one-stent group. In this context, we should emphasise that our study is not intended to compare the specific merits of the different stent techniques, or to attack one of them. It just concludes that if, for whatever reason or consideration by the operator or Heart Team, a one-stent technique can be tried as first option, this is preferable. It does explicitly not exclude that in some lesions a two-stent technique might perform better. But, wherever it is possible to keep it simple, it should be kept simple.

Despite these limitations, we believe that this retrospective analysis is representative for bifurcation stenting in every day practice.

## Conclusion

In conclusion, this study shows that in our series there is no difference in restenosis rates between provisional stenting compared with two-stent techniques for bifurcation stenting, taking into account that more complex lesions may more often warrant a two-stent technique. Therefore, since provisional stenting is the simplest, most straightforward and cheapest approach, if technically feasible this technique has our preference as the initial approach and an upgrade can be considered if an insufficient result is obtained.
